# The Interconnected World of Coagulation Factors, Platelets and Plasminogen: A Novel Perspective on Biosynthetic Regulation

**DOI:** 10.3390/life15101593

**Published:** 2025-10-13

**Authors:** Ivan Bergo, Mark Slevin, Ylenia Pastorello, Aaron Höhne, Amelia Tero-Vescan

**Affiliations:** 1 Faculty of Medicine in English Campus Hamburg, George Emil Palade University of Medicine, Pharmacy, Science and Technology, 540139 Târgu Mureș, Romania; ivanchitarra@gmail.com (I.B.); aaron2001@me.com (A.H.); 2 Center for Advanced Medical and Pharmaceutical Research (CCAMF), George Emil Palade University of Medicine, Pharmacy, Science and Technology, 540139 Târgu Mureș, Romania; 3 Department of Anatomy and Embryology, George Emil Palade University of Medicine, Pharmacy, Science and Technology, 540139 Târgu Mureș, Romania; ylenia.pastorello@gmail.com; 4 Department of Biochemistry, George Emil Palade University of Medicine, Pharmacy, Science and Technology, 540139 Târgu Mureș, Romania; amelia.tero-vescan@umfst.ro

**Keywords:** platelet-derived mediators, thrombopoietin regulation, fibrinogen degradation products, interleukin-6 signaling, plasminogen biosynthesis

## Abstract

Platelets play a pivotal role in coagulation, traditionally recognized for their involvement in thrombin generation via the prothrombinase complex and for regulating thrombopoietin (TPO) synthesis through platelet-mediated TPO uptake. However, recent studies suggest that TPO homeostasis involves more dynamic, feedback-driven mechanisms, though these interactions remain incompletely described and experimentally confirmed. The interplay between platelet activating factor (PAF) secretion, fibrinolysis, interleukin-6 (IL-6) signalling, hepatic TPO synthesis, as well as the complexity of platelet subpopulations, emphasises platelets’ multifaceted role in haemostasis and haematopoiesis. Our article investigates novel pathways by which fibrinogen degradation products (FgDPs) influence plasminogen and TPO synthesis, focusing on the interconnection between procoagulant platelets, platelet-derived messengers, and fibrinolytic processes. In this work several intermediary mechanisms are hypothesised, including the FgDP-IL-6-plasminogen pathway, the PAF-IL-6-TPO pathway, and the thrombin-FgDP-IL-6-TPO pathway, which may link FgDP and plasminogen biosynthesis with platelet activation, cytokine release, and thrombopoiesis regulation. The proposed mechanisms involve secretion of PAF by procoagulant platelets, inducing IL-6 synthesis in endothelial cells, fibroblasts, and vascular smooth muscle cells. Subsequently, IL-6 stimulates hepatocyte-driven TPO production, potentially serving as a feedback mechanism to restore platelet counts following coagulation. Furthermore, fibrinolysis-generated FgDPs may further enhance IL-6 release, implying alternative routes for TPO regulation. Our hypotheses challenge the prevailing view that platelet numbers alone dictate TPO homeostasis. Therefore, we propose that inflammatory and fibrinolytic signals actively regulate TPO homeostasis, challenging the platelet-count-centric paradigm. These insights provide a new perspective on haematopoiesis and suggest novel therapeutic targets for thrombocytopenia and coagulation disorders, highlighting the need for further experimental validation.

## 1. Introduction: Haemostatic Regulation of the Coagulation Pathway and Coagulation Factor Dynamic Storage and Turnover

**Figure 1:**
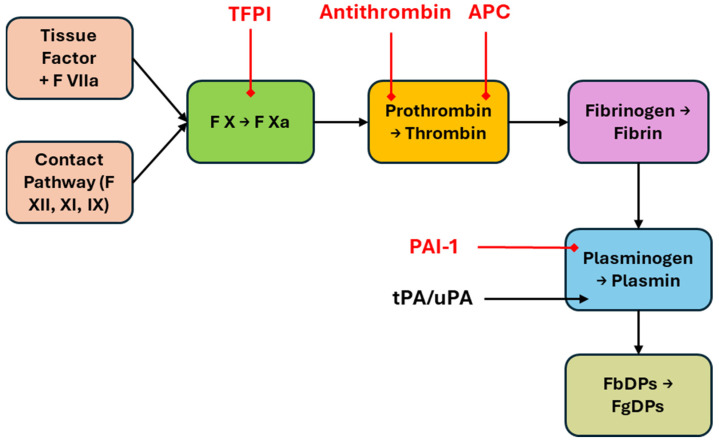
Classical coagulation and fibrinolysis pathways. The extrinsic and intrinsic coagulation pathways converge on factor X activation, leading to thrombin generation, fibrin formation, and clot stabilization via factor XIII. Fibrinolysis is mediated by conversion of plasminogen to plasmin by tissue plasminogen activator (tPA) or urokinase-type plasminogen activator (uPA). Plasmin degrades fibrin into fibrin degradation products (FbDPs) and fibrinogen degradation products (FgDPs). Key inhibitory checkpoints include antithrombin, tissue factor pathway inhibitor (TFPI), activated protein C (APC), and plasminogen activator inhibitor-1 (PAI-1). Solid arrows indicate established biochemical pathways. Red arrows indicate inhibition.

Three major and overlapping/common pathways are involved in the regulation of coagulation factor replenishment. These are primarily associated with (1) thrombin–prothrombin cycle regulated feedback, (2) fibrinogen degradation regulated feedback and (3) coagulopoietin feedback. Whilst the first two pathways regulate de novo synthesis of different coagulation factors, the last pathway, id est, the coagulopoietin pathway, regulates the biologic activity of different coagulation factors, without directly affecting the synthesis rate [1]. The coagulation and fibrinolysis systems form two tightly regulated cascades that balance clot formation and dissolution. The coagulation cascade is initiated either by exposure of tissue factor (extrinsic pathway) or by contact activation (intrinsic pathway), both converging on the activation of factor X and subsequent thrombin generation [2,3]. Thrombin converts fibrinogen to fibrin, activates factor XIII to crosslink fibrin, and amplifies its own generation through feedback activation of upstream cofactors [1,4]. Fibrinolysis is initiated when tissue plasminogen activator (tPA) or urokinase-type plasminogen activator (uPA) convert plasminogen to plasmin, which degrades fibrin into soluble fibrin degradation products (FbDPs) and fibrinogen degradation products (FgDPs) [5,6,7]. Regulation occurs through inhibitors such as antithrombin, tissue factor pathway inhibitor (TFPI), protein C, and plasminogen activator inhibitor-1 (PAI-1), ensuring clot stability yet allowing eventual resolution [8,9,10,11]. These canonical cascades have been extensively characterized and are summarized in Figure 1. On this foundation, our review focuses on specific feedback circuits, particularly those linking FgDPs, platelet-derived mediators, and IL-6, to highlight underappreciated mechanisms that may influence thrombopoiesis.

### 1.1. Prothrombin Pathway

Identification that the prothrombin pathway was responsible for controlling the rate of synthesis of coagulation factor X and prothrombin was described as early as 1981 by Graves et al. and 1990 by Mitropoulos and Esnouf [4,5]. The transition of prothrombin to thrombin, catalysed by prothrombinase, represents the only non-redundant step in thrombus formation and therefore represents a critical regulatory end point in the coagulation pathway. Once prothrombin is split to form thrombin, other important degradation products, such as prothrombin fragment 1.2, are concomitantly formed. Of significant interest was the finding also by Mitropoulos and Esnouf, that the prothrombin fragment 1.2 (PF1.2), was an important component of prothrombin and, that once generated, it migrated into the interstitial fluid where part of it bound local protease-activated receptors (PAR1-3) [5].

Graves et al. in 1981 [4] first demonstrated the importance of this product characterising a γ-carboxyglutamyl residue-dependent transition that was essential for the regulation of synthesis to take place (more recently Gla-9 residue being the most prominent isomer) [6]. They went on to demonstrate PF1.2-induced and dependent synthesis of both coagulation factor X and prothrombin, indicating that it was acting through a feedback mechanism. It is important to know that the response of the body towards increasing levels of prothrombin degradation was dose dependent and saturable based upon reaching binding equilibrium. These studies were carried out using H4-11-E-C3 rat hepatoma cells (used because they retain liver-specific functions and respond to IL-6 with upregulation of acute-phase genes, including TPO) and a strong correlation was observed on pathway activation even in the absence of leukocytes, suggesting a direct regulatory mechanism [4]. That being said, to date detailed investigations into the saturable nature of their clearance or the existence of specific binding equilibria are limited, whilst the importance of understanding the kinetics prothrombin activation and degradation, as well as the role of degradation products in coagulation and inflammation was recently and eloquently described by Tsantes et al. [7], who encapsulated the interplay between coagulation regulation and inflammation, focussing on FgDPs and D-dimer (an FbDP) as critical IL-6 modulators informing of the pathophysiological progression in sepsis and related conditions.

It was more than two decades later before more detailed solutions to the unanswered questions about both enzymological and structural binding analysis identified other key regulatory elements. For example, the critical importance of cofactors such as Va binding to Xa to enable prothrombinase complex formation, with vitamin K dependence; the dissociation of PF1.2 from thrombin allowing its membrane release and circulation [8]; cleavage of prothrombin by prothrombinase via Arg320 followed by Arg271 (a process known as ratcheting), yields a zymogen-like form of meizothrombin IIa an intermediate that determines thrombin production which is now considered as the physiological pathway [9,10]; and the importance of the 10 Gla residues in the prothrombin-Gla domain plays an important role as a calcium-dependent anchor to interact with negatively charged phospholipids, leading to the formation of a prothrombinase complex in the presence of vitamin K, so loss of Gla residues decreases prothrombin activity and thrombin formation [11]. This pathway, when considered in isolation, is therefore relatively well characterized to date.

### 1.2. Fibrinogen Metabolism

It is important to distinguish between fibrinogen degradation products (FgDPs) and fibrin degradation products (FbDPs). FgDPs arise when plasmin cleaves soluble fibrinogen, generating fragments such as X, Y, D, and E, and therefore reflect fibrinogenolysis. By contrast, FbDPs are produced when plasmin degrades crosslinked fibrin within a thrombus. D-dimer, the most clinically relevant degradation fragment, is generated only from crosslinked fibrin and thus represents an FbDP rather than an FgDP. Its presence therefore indicates that thrombin activity, fibrin polymerization, crosslinking by factor XIIIa, and subsequent plasmin-mediated fibrinolysis have all occurred. Synthesis, activation and degradation components of this pathway are integrally responsible for regulating the systemic circulating levels of fibrinogen. Once a clot has formed, the plasmin system will be activated leading to the resolution of the clot by degrading fibrin in fibrin degradation products (FbDPs). The plasmin system however will not only affect the fibrin, forming FbDP, but also the fibrinogen, forming FgDP. The role of FgDP in stimulating fibrinogen synthesis is confirmed in both sources [12] and [13], while the role of FbDP is more debated. Regardless of the pathway, these degradation products activate leukocytes, prompting them to secrete hepatocyte-stimulating factor (HSF), now identified as interleukin-6 (IL-6) [14], which subsequently induces hepatocytes to synthesize fibrinogen [13,15]. The synthesis of fibrinogen can be enhanced by glucocorticosteroids (dexamethasone) synergising with IL-6 when applied to rat hepatocytes [16]. More recently, fragment E derived from fibrin/fibrinogen has been shown to stimulate the production of inflammatory cytokines via the p150,95 integrin on macrophages, highlighting its pro-inflammatory potential [14]. Furthermore, glucocorticoids have been found to enhance IL-6-dependent γ-fibrinogen expression by interfering with the expression of the suppressor of cytokine signalling 3 (SOCS3), thereby prolonging STAT3 activation and promoting acute-phase gene induction in hepatocytes [17,18].

Leukocytes seem to be sufficient to elicit this action, and an inflammatory environment does not appear to be necessary as in both cases the stimulation of fibrinogen synthesis still occurred without the need of inflammation. For example, FgDPs were induced by intravenous (IV) injection of fibrinogenolytic and fibrinolytic substances in test subjects, without any underlying inflammation (with the exception of wound healing taking place at the insertion site) [12] and in the other in vitro study even though leukocytes were present, an inflammatory stimulus was not [13]. Other studies have further elucidated the role of FgDPs in stimulating fibrinogen synthesis. LaDuca et al. [19] demonstrated that FgDP fragment D (but not fragment E) directly and specifically enhances fibrinogen production in cultured rat hepatocytes, independent of additional serum factors. Similarly, Moshage et al. [20] found that FgDPs increase fibrinogen synthesis in rat liver by elevating fibrinogen mRNA levels. These findings support the concept that FgDPs can directly influence fibrinogen synthesis without necessitating an inflammatory environment (meaning no systemic biomarker-based evidence of inflammatory hyperactivity nor equivalent local micro-environmental tissue activity) [21].

Despite solid evidence supporting the role of FgDPs in stimulating fibrinogen biosynthesis under physiological conditions, the role of IL-6 remains less clear. While IL-6 undoubtedly contributes to increased fibrinogen levels in pathological states, its role in physiological conditions is still debated.

In a population-based study, Cronjé et al. [22] investigated the relationship between IL-6 and fibrinogen levels, including the γ’ isoform, in 201 apparently healthy black South African individuals from the PURE (Prospective Urban and Rural Epidemiological) study. Using ELISA assays, the researchers quantified plasma concentrations of IL-6, total fibrinogen, and γ’ fibrinogen, while also genotyping several fibrinogen gene polymorphisms (FGA rs6050, FGB rs1800790, FGG rs2066865). They found that IL-6 was positively associated with both total fibrinogen and γ’ fibrinogen, supporting the cytokine’s role as a regulator of acute-phase protein synthesis. Moreover, the influence of IL-6 on fibrinogen levels was modulated by the presence of specific genetic variants, suggesting a gene-cytokine interaction in the regulation of fibrinogen expression and isoform distribution.

Rein-Smith et al. [23] conducted an in vitro study using HepG2 human hepatocellular carcinoma cells (derived from a well-differentiated hepatocellular carcinoma in a 15-year-old Caucasian male, it is widely used as a hepatocyte model because it retains liver-specific functions and shows IL-6–responsive expression of acute-phase proteins, including fibrinogen and TPO) to assess the direct effect of IL-6 on fibrinogen gene expression. Cells were stimulated with recombinant IL-6, and fibrinogen mRNA isoforms were measured by quantitative reverse transcription-PCR (qRT-PCR). The study demonstrated that IL-6 significantly increased total fibrinogen expression, with a 3.6-fold rise in γA mRNA and a notably higher 8.3-fold increase in γ’ mRNA, indicating a preferential induction of the γ’ isoform. This result implies that IL-6 not only enhances fibrinogen synthesis overall but also alters the isoform composition, which may impact the structure and function of fibrin clots, particularly in prothrombotic conditions. Together, these studies highlight both the inflammatory and genetic regulation of fibrinogen synthesis, especially the production of its γ’ variant.

FgDP-induced biosynthesis has been shown not to affect albumin biosynthesis. However, IL-6, as a proinflammatory cytokine, reduces albumin biosynthesis. This discrepancy in the regulation of albumin biosynthesis has led earlier studies to question IL-6’s role as a physiological mediator of fibrinogen biosynthesis [20]. A recent study, however, demonstrated reduced fibrinogen biosynthesis in IL-6 receptor knockout HepG2 cells, even in the absence of IL-6 in the surrounding medium. They further identified other regulatory genes including LEPR and PDIA5 whose knockout promoted fibrinogen synthesis confirming a complex genetic regulatory pathway exists. In any case, theirs and other results suggest that HepG2 cells, and possibly hepatocytes (since this cell line is in fact a tumor cell line), are highly sensitive to IL-6, requiring only extremely low levels of the cytokine to induce fibrinogen biosynthesis. It can therefore be argued that the IL-6 levels required for fibrinogen biosynthesis are so low that they do not significantly impact albumin biosynthesis [24]. In summary, we highlight the importance of gaining a greater understanding of the regulation of fibrinogen gene expression in hepatocytes/the liver, and the potential implications for diseases associated with abnormal fibrinogen levels.

#### Physiological Versus Pathological Roles of IL-6 in Fibrinogen Regulation

IL-6 plays a dual role in the regulation of fibrinogen biosynthesis, depending on whether its action occurs in a physiological or pathological setting. Under physiological conditions, IL-6 functions as a hepatocyte-stimulating factor that maintains fibrinogen homeostasis. Very low levels of IL-6 are sufficient to enhance fibrinogen transcription in hepatocytes [23,24], supporting a transient increase in fibrinogen during acute tissue injury or infection. This adaptive acute-phase response helps restore haemostasis without markedly impairing albumin synthesis [20,24].

In contrast, pathological IL-6 elevations, such as in chronic inflammation, autoimmune disorders, atherosclerosis, and cancer, drive sustained fibrinogen upregulation, including an overrepresentation of the γ′ isoform, which is associated with a prothrombotic phenotype [22,23]. Persistently elevated IL-6 can also stimulate hepatic thrombopoietin (TPO) synthesis, promoting reactive thrombocytosis and further linking inflammation to thrombotic risk [25].

In sepsis and other hyperinflammatory states, IL-6 surges are extreme, leading to marked fibrinogen overproduction alongside albumin suppression, creating a hypercoagulable yet hypoalbuminemic state [7]. Importantly, fibrinogen degradation products (FgDPs) can further amplify this process by stimulating IL-6 release from monocytes and hepatic tissue [14,15,26], establishing a vicious feed-forward loop between fibrinolysis and inflammation. This maladaptive circuit contributes to disseminated intravascular coagulation (DIC) and thromboinflammatory complications. From a translational standpoint, IL-6 inhibitors such as tocilizumab may therefore have therapeutic relevance in limiting aberrant fibrinogen and TPO upregulation in sepsis and other thromboinflammatory conditions [27,28].

### 1.3. Coagulopoietin Pathway

In this review we use the term “coagulopoietin” to denote a hypothetical class of mediators derived from coagulation or fibrinolysis that signal to the liver to regulate TPO synthesis, thereby linking haemostatic activity to platelet production. This is the least understood of the coagulation regulation mechanisms. While its underlying premise is straightforward, a major challenge lies in the lack of information regarding key aspects of its function. Coagulopoietins are a group of heat-resistant endogenous factors released in response to a decrease in the biological activity of specific coagulation factors.

Currently, our team has identified, within the existing literature, two coagulopoietins: coagulopoietin-II and coagulopoietin-X. These endogenous factors are commonly found in animals treated with coumadin, an anticoagulant that reduces the biological activity of vitamin K-dependent coagulation factors. Since vitamin K is essential for their optimal function, coagulopoietins appear to enhance the biological activity of these factors without affecting their synthesis rate. The precise mechanism by which this occurs remains unknown, but it is suspected to involve the complete carboxylation of the molecule [1]. However, several critical questions remain unanswered, for example, the source of secretion—the specific organs or cell types responsible for secreting coagulopoietins have not yet been conclusively identified; and the specificity—it is unclear whether coagulopoietins exist for all coagulation factors or are exclusive to vitamin K-dependent ones. Although the identity of the secreting cells remains unclear, it is plausible that hepatocytes, as the main site of synthesis of vitamin K-dependent coagulation factors, may also play a role in releasing coagulopoietin-like regulators. Alternatively, monocyte–macrophage lineages and endothelial cells, which are central players in the inflammatory–haemostatic interface, represent potential sources, either through acute-phase responses or in reaction to reduced factor activity. The possibility of multiple tissue origins cannot be excluded, and delineating these cellular contributors remains an important future research direction.

In this regard, Hao et al. [29] developed a mammalian cell-based assay using HEK293 cells to study the γ-carboxylation of vitamin K-dependent coagulation factors, with a particular focus on factor IX. They engineered cells to express coagulation factor precursors and measured γ-carboxylation activity through a GLA domain-specific ELISA and mass spectrometry. The study showed that the propeptide region of factor IX is essential for substrate recognition by γ-glutamyl carboxylase (GGCX), the enzyme responsible for vitamin K-dependent carboxylation. Mutations in the propeptide impaired carboxylation, even when vitamin K was abundant. These findings support the idea that complete γ-carboxylation of glutamic acid residues, rather than protein abundance, governs the biological activity of coagulation factors and explain the warfarin hypersensitivity observed in individuals with certain propeptide mutations. This platform also provides a high-resolution model for testing how genetic or pharmacological factors affect coagulation activity at the post-translational level.

Summarising recent and historical knowledge within the field, Girolami et al. [30], eloquently described a comprehensive analysis of the structure, function, and clinical significance of vitamin K-dependent coagulation factors, including factors II, VII, IX, and X, as well as protein C and protein S. The evidence confirms that vitamin K is crucial for the γ-carboxylation of specific glutamic acid residues in the GLA domain, a modification necessary for calcium binding and membrane association, key steps in coagulation cascade activation. Importantly, the review highlighted that defective γ-carboxylation, due to genetic mutations (e.g., in the GGCX or VKORC1 genes) or vitamin K antagonists (like warfarin), can lead to either bleeding disorders (via reduced activity) or thrombotic risk (via overcarboxylated or gain-of-function mutants). The authors noted that mutations in the prothrombin (F2) and factor IX (F9) genes can result in paradoxical hypercoagulability, even when antigen levels are normal, further supporting the idea that post-translational quality, not just quantity, determines coagulant activity.

Comprehensive research remains essential to fully elucidate the role and mechanisms of the coagulopoietin pathway, its implications in coagulation regulation, and therapeutic applications. Moreover, the precise mechanisms in which coagulopoietins enhance the activity of their target factors and their overall function in coagulation are subjects of ongoing investigation.

## 2. Overview of the Coagulation Pathway and Control of Inflammation During Insult and Injury: Known Determinants and Their Role or Function

Haemostasis, the process through which blood loss is prevented after tissue insult, is governed by the coagulation cascade and its pathways, eventually leading to its final product, thrombus formation. Damage to endothelial tissue, following vascular trauma, causes the activation of tissue factor (TF), a 44,000 Da molecular weight membrane-bound glycoprotein synthesized in the vascular adventitia, which binds to factor VIIa, creating the TF–VIIa complex. The TF–VIIa complexes further activate Factor IX and X, therefore prompting the proteolytic cascade resulting in thrombin and stable fibrin clot formation [31,32].

Pro-inflammatory cytokines, namely Tumour Necrosis Factor (TNF), Interleukin-1 alpha (IL-1α), Interleukin-1 beta (IL-1β), Interleukin-6 (IL-6), Interleukin-8 (IL-8), Interferon-gamma (IFN-γ), and Monocyte Chemoattractant Protein-1 (MCP-1), increase in vitro TF expression on endothelial cells (ECs) [32]. This crosstalk between inflammation and coagulation extends further, as additional acute-phase proteins such as C-reactive protein (CRP) also converge on the regulation of TF expression. The acute-phase pentameric CRP has been linked to upregulation of TF expression in ECs, promoting their pro-atherothrombotic phenotype and proliferation, in a dose-dependent fashion, through mediation by activation of the p44/42 MAP Kinase (ERK 1/2) pathway [33]. Platelets represent vital mediators of the inflammatory response, and they are formed and activated during inflammatory conditions by chemokines binding to their surface receptors, a process that induces a procoagulant state. Activated platelets display the ability to interact with CRP and accelerate dissociation into its highly biologically active monomeric subunits (mCRP), therefore dramatically contributing to dissemination of inflammation [34].

**Figure 2:**
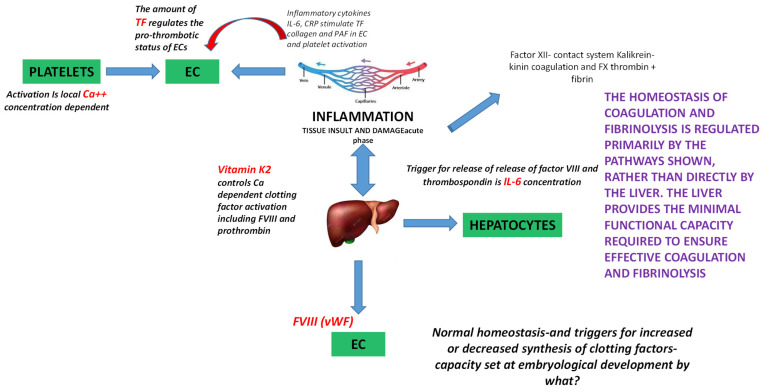
Critical intermediates of the coagulation-response to injury cascade—the knowns and the unknowns. This figure summarizes the current (partially hypothesized) knowledge to date and shows ‘complex’ regulation of coagulation factors from an inflammatory perspective, starting from the injury which induces both the activation of the immune system and the coagulation and aggregation system. Injury activates platelets, by increasing intracellular levels of Ca^2+^. The activation is concentration-dependent, i.e., the higher the Ca^2+^ levels the higher the activation. Activated platelets will then induce the pro-thrombotic state of ECs by reacting with them and increasing TF expression and synthesis. Concomitantly inflammation will induce the pro-thrombotic status of EC by increasing TF levels. The expression of TF and its synthesis are controlled by increasing the levels of the pro-inflammatory cytokines IL-6 and CRP. These cytokines can also induce collagen synthesis and activate platelets through PAF. There is also interplay between the inflammatory site and the liver. The inflammatory site leads to the synthesis of positive acute phase proteins and inhibits the synthesis of negative acute phase proteins through the synthesis and release of cytokines into the bloodstream. Among these many proteins, FVIII and thrombospondin are released by increased levels of IL-6. Vitamin K2 is also important for the activation of FVIII and prothrombin (highlighted in red). Many elements described within the figure represent areas that require further investigation in order to obtain a more complete understanding of the coagulation pathway. Created with Microsoft PowerPoint. Legend: C-reactive protein (CRP), calcium ions (Ca^2+^), endothelial cells (ECs), factor VIII (FVIII), factor X (FX), interleukin-6 (IL-6), platelet activating factor (PAF), tissue factor (TF).

The pro-inflammatory and pro-thrombotic actions of CRP also intersect with other endothelial-derived haemostatic proteins. Following vascular insult of any aetiology, von Willebrand factor (VWF) is released into the plasma, where it acts as carrier of FVIII and “protector” of its degradation. These two factors, directly produced by ECs, play a crucial role in the haemostatic system, namely in platelet and subendothelial collagen adhesion and in proteolytic activation of factor X by factor IXa, respectively [2,35]. Recently, VWF and FVIII have been described as possible acute-phase reactants and biomarkers of inflammation, due to their elevation during infection, but also in chronic inflammatory conditions, such as autoimmune disorders. The resulting procoagulant state associated with the inflammatory milieu has been defined as “thromboinflammation” [2,3]. Hence, haemostatic balance is strictly dependent on the inflammatory micro-environment. However, unanswered questions remain, particularly in deciphering the master controls that determine the absolute outcome (Figure 2).

The components discussed in this review have been purposefully selected to illustrate specific, functionally relevant regulatory circuits within the haemostatic and coagulation cascade. Rather than presenting an exhaustive survey of all cellular and molecular elements involved in haemostasis, the review aims to trace how discrete but interlinked events, such as fibrinogen cleavage, fibrin polymerization, generation of fibrin(ogen) degradation products, and their downstream signalling effects, participate in a coordinated network that extends beyond clot formation. We have formulated this section from documented biochemical interrelationships, such as the capacity of FgDPs to induce cytokine release, or the feedback between platelet activity and hepatocyte-driven acute-phase responses. This focused approach has enabled us to develop a mechanistic exploration of how specific molecular events contribute to dynamic regulation within the haemostatic system, including both procoagulant and modulatory (e.g., inflammatory) outcomes. While the proposed pathways may be conceptual in parts, they are based on established interactions and are intended to create further testable hypotheses that can inform future experimental inquiry. Thus, the review consolidates these particular (discrete) haemostatic elements into a unified functional perspective, highlighting what we believe are highly underappreciated regulatory mechanisms within the broader coagulation context.

## 3. Defining the Critical (Overlapping) Factors: State of the Art and Future Perspectives

### 3.1. Regulation of Plasminogen Levels

Plasminogen is an acute-phase protein, and studies have shown that IL-6 increases plasminogen mRNA expression and elevates plasma plasminogen levels [36,37,38]. Based on this knowledge we can assume that the regulation of plasminogen levels is primarily influenced by IL-6 stimulation, which occurs when monocytes or macrophages are activated by FgDP (and possibly FbDP). Although there is no consensus on which specific fibrinogen fragment induces IL-6 release, all previously published studies agree that FgDPs play a significant role in this process [13,15,26].

Before discussing this hypothesis, it is essential to summarize the basic mechanism of fibrinolysis. Notably, high-affinity receptors for thrombin are present on ECs [39]. Fibrinolysis is initiated by thrombin-dependent release of tPA from ECs [40]. However, tPA requires fibrin to be fully effective, as fibrin sequesters tPA from circulation and protects it from inhibition by plasminogen activator inhibitor-1 (PAI-1) [41,42]. The mechanism behind thrombin-dependent tPA release has been extensively studied, revealing that tPA release occurs approximately six hours after thrombin stimulation [40].

For tPA release to occur, thrombin must provide more than just proteolytic activity. According to the “two-signal hypothesis,” thrombin requires both an intact catalytic site and the ability to bind to a specific high-affinity endothelial receptor. This dual requirement ensures that thrombin’s action is spatially and temporally restricted: the catalytic activity generates fibrin, while receptor binding localizes thrombin to endothelial cell surfaces where it can trigger regulated tPA secretion. Without receptor engagement, catalytically active thrombin alone is insufficient to stimulate tPA release, highlighting the importance of coordinated enzymatic activity and receptor-mediated signaling in linking coagulation to fibrinolysis [43]. Thrombin also appears to increase cyclic adenosine monophosphate (cAMP), stimulate phosphoinositide turnover, and induce diacylglycerol (DAG) release. DAG metabolites subsequently activate protein kinase C (PKC), which, along with other intracellular mediators, stimulates tPA biosynthesis. However, PAI-1 is also released along with tPA [43,44].

Although human protein C (PC) and activated protein C (APC) were initially proposed to mediate thrombin-induced tPA release [40], this is unlikely. APC, an anticoagulant activated by thrombin, inhibits thrombin-induced PAI-1 release and lowers PAI levels by forming complexes with them, [45,46]. Some studies supporting APC as a secondary messenger for tPA release used bovine APC, which is more effective than human APC at clearing PAI from circulation. APC nevertheless contributes to fibrinolysis indirectly: it inactivates PAI-1, the main inhibitor of tPA, enhancing tPA activity; and it suppresses thrombin generation by inactivating factors Va and VIIIa, reducing TAFI activation and further promoting fibrinolysis (reviewed in [47,48]). Additionally, studies on human APC in spider monkeys failed to show an increase in tPA levels [46]. Given that bovine APC appeared to increase tPA levels while reducing PAI, we suspect that the observed increase in tPA was not due to APC-induced tPA synthesis but rather a decrease in PAI levels, which prevented PAI from binding to tPA. This lack of human APC efficacy in removing PAI from circulation explains why no increase in tPA was observed in the spider monkey study. Studies examining activated protein C (APC) show marked interspecies differences in its ability to modulate fibrinolysis. In bovine models, APC has been reported to increase tPA release and enhance fibrinolysis, whereas in humans APC fails to elicit such an increase [39,40]. Data from non-human primates, such as spider monkeys, appear more consistent with the human phenotype, where APC does not significantly upregulate tPA [41]. These findings highlight that results from bovine or rodent models cannot be directly extrapolated to human pathophysiology. Nevertheless, the convergence of human and primate data suggests that in humans the dominant antifibrinolytic effect of APC is mediated via suppression of plasminogen activator inhibitor-1 (PAI-1) rather than stimulation of tPA release. This distinction is important for translational relevance: while bovine studies imply a dual mechanism of action, in humans APC appears to act primarily by removing the PAI-1 brake on fibrinolysis. Thus, although species differences caution against simplistic translation, the primate and human data support the plausibility of our hypothesis that APC exerts a regulatory role in human fibrinolysis mainly through modulation of PAI-1 activity rather than by increasing tPA production.

In summary, fibrinolysis is primarily regulated by thrombin, tPA and fibrin, while APC plays a secondary role by reducing PAI levels.

#### Proposed Mechanism: The FgDP–IL-6–Plasminogen Pathway

With the foundational knowledge established, we now present our hypothesis. It is important to emphasize that the following proposed mechanism remains hypothetical and has not yet been empirically validated through direct experimentation. Additionally, to the best of our knowledge, no prior publications have explicitly described this pathway. While this concept is novel, it is theoretically grounded in well-established biological principles and should be considered a logical extension and an innovative perspective on existing scientific understanding.

**Figure 3:**
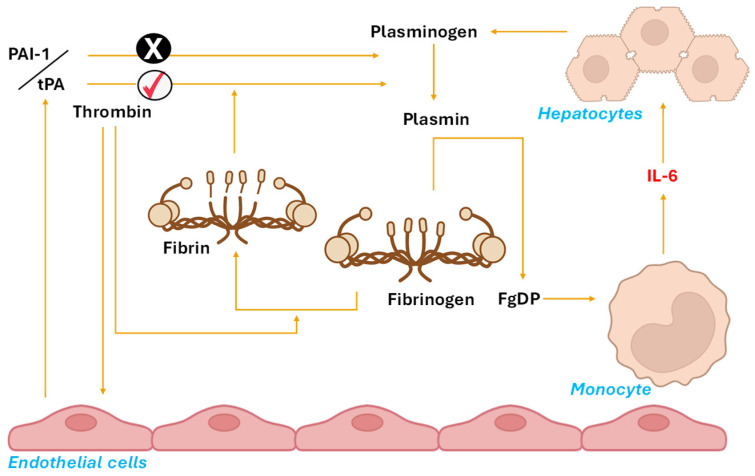
Visual representation of the FgDP–IL6–plasminogen pathway. Thrombin and plasmin lead to fibrin and FgDP formation respectively. FgDP formation requires plasmin, which is activated by tPA and inhibited by PAI-1. The tPA release by itself is controlled via thrombin. FgDPs then act on monocytes to stimulate IL-6 which increases plasminogen synthesis in hepatocytes. Created with BioRender. Legend: fibrinogen degradation products (FgDPs), interleukin 6 (IL-6), plasminogen activator inhibitor-1 (PAI-1), tissue plasminogen activator (tPA), ✘ means inhibitor effect and ✔ means activator effect.

In the FgDP–IL-6–plasminogen pathway, thrombin stimulates the release of tPA and promotes fibrin formation. In the presence of fibrin, tPA activates plasmin, which subsequently degrades both fibrin and fibrinogen, forming FbDP and FgDP respectively. These FgDPs activate monocytes, triggering the synthesis of IL-6, which in turn stimulates hepatocytes to release plasminogen, replenishing its plasma levels. This proposed pathway is illustrated in Figure 3. In our model we have emphasized FgDPs, particularly fragment D and D-dimer, because these species are the most consistently shown to stimulate IL-6 release in monocytes and hepatic tissue [14,15,26]. In contrast, the role of FbDPs in IL-6 regulation remains less well established. Some early studies suggest that FbDPs, especially fragment E, can exert immunomodulatory effects, such as influencing macrophage activation and lymphocyte proliferation, but their ability to induce IL-6 appears to be more limited and context dependent [14]. Indeed, Mandl et al. demonstrated that fragment D but not fragment E significantly increased IL-6 output in perfused mouse livers [15], while Lee et al. reported IL-6 induction by fragment E in rat macrophages, underscoring cell-type and species variability [14]. These findings indicate that FbDPs may contribute to cytokine signalling under certain conditions, but compared to FgDPs their impact on IL-6 pathways is weaker, inconsistent, or restricted to specific cellular contexts. Therefore, for the purpose of linking fibrinolysis to hepatic thrombopoietin synthesis, FgDPs are likely to be the predominant mediators, while FbDPs may play at most a minor or modulatory role without fundamentally altering the proposed feedback loops.

### 3.2. Regulation of Platelets Levels

Early research on platelet involvement in coagulation established that platelets play a crucial role in thrombin formation via the prothrombinase complex on procoagulant platelets [49]. The regulation of platelet levels is primarily controlled by thrombopoietin (TPO), a hormone synthesized in the liver, which induces hematopoietic stem cells to differentiate into megakaryocytes, the precursors of platelets [50]. Initially, TPO regulation was thought to occur solely through platelet-mediated uptake, where excess platelets reduce circulating TPO levels, while a deficiency leads to increased TPO availability [51].

Subsequent studies challenged the idea that platelet uptake is the only mechanism regulating TPO levels. New findings of experiments conducted in mouse models (where desialylated or senescent platelets engaged hepatocyte AMR and increased hepatic TPO mRNA and plasma TPO levels) and in vitro (in hepatocyte cultures where desialylated platelets or glycoproteins triggered AMR-dependent JAK2–STAT3 signaling) demonstrated that aged platelets can stimulate TPO production via a receptor-mediated mechanism involving the Ashwell-Morell Receptor (AMR) on hepatocytes [51,52]. When desialylated platelets bind to AMR, they are endocytosed, triggering increased TPO mRNA transcription and subsequent TPO synthesis, concomitantly providing a feedback loop where the clearance of aged platelets directly influences the production of new platelets via TPO synthesis. This mechanism shares similarities with IL-6 receptor activation, involving JAK2-STAT3 phosphorylation and nuclear translocation [52]. In parallel, studies confirmed that IL-6 can directly stimulate hepatic TPO synthesis [25,51,53,54]. Further investigations showed that after partial hepatectomy in mice, the AMR and IL-6 receptor (IL-6R) pathways synergistically activated the JAK2-STAT3 signaling cascade, leading to increased TPO production. This response was crucial for restoring platelet counts following liver injury [55]. Building upon these insights, our team proposes an additional regulatory mechanism: the platelet activating factor–IL-6–TPO pathway (PAF–IL6–TPO pathway). It is increasingly clear that platelet subpopulations exert distinct effects on cytokine signalling and TPO regulation. Aggregatory platelets primarily mediate thrombus formation and clot retraction, with limited impact on IL-6 release. By contrast, procoagulant platelets undergo necrotic transformation and secrete PAF as well as proinflammatory microparticles [49,56,57,58]. PAF strongly induces IL-6 synthesis in endothelial cells, fibroblasts, and vascular smooth muscle cells [59,60,61,62], thereby linking procoagulant platelet activity to hepatic TPO upregulation. This subpopulation-specific behaviour suggests that platelet-mediated control of thrombopoiesis is context-dependent, being minimal under physiological haemostasis but amplified during thromboinflammatory conditions.

#### 3.2.1. Proposed Mechanism: The PAF–IL-6–TPO Pathway

**Figure 4:**
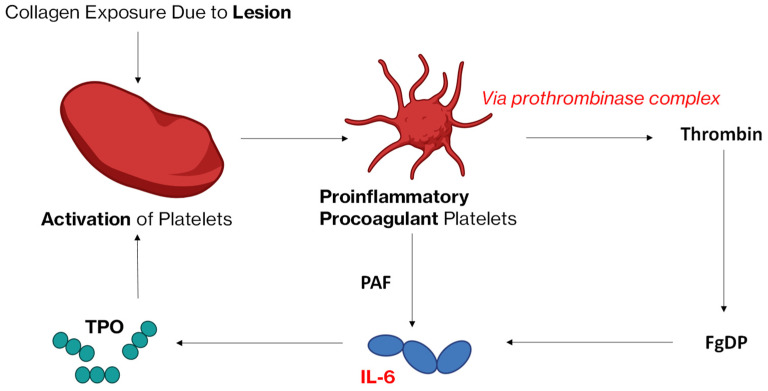
A visual representation of the PAF–IL-6–TPO pathway. Following an endothelial lesion, collagen exposure will cause activation of the procoagulant platelets. This will stimulate the release of two compounds: one being PAF and the second one being thrombin. The latter utilizes prothrombinase complexes and leads to thrombus formation. Fibrinolysis will then also lead to the formation of FgDP. FgDP and PAF will then stimulate the secretion of IL6, which in turn stimulates the release of TPO. This hormone further induces thrombopoiesis. Created with BioRender. Legend: fibrinogen degradation products (FgDPs), interleukin-6 (IL-6), platelet activating factor (PAF), thrombopoietin (TPO). Note, Experimental evidence supports the plausibility of this pathway, as FgDPs induce IL-6 secretion in monocytes and liver tissue [14,15,26], while IL-6 in turn upregulates hepatic TPO synthesis in vitro and in vivo [25,54].

Recent findings categorized activated platelets into procoagulant platelets and aggregatory platelets, with procoagulant platelets undergoing necrosis and secreting PAF [49,56]. PAF secretion has been linked to platelets activated by collagen, thrombin, and shear stress, and is primarily transported in circulation via microparticles [57,58]. Since PAF has been shown to induce IL-6 secretion in ECs, vascular smooth muscle cells, and fibroblasts [59,60,61,62], we hypothesize that PAF released from procoagulant platelets stimulates IL-6 production, which in turn increases hepatic TPO synthesis to compensate for platelet loss. Further studies strengthen this argument, for example, the finding that PAF, via the PAF-receptor, enhances IL-6 production through the activation of NF-κB (promoting inflammation), and via protein tyrosine phosphatase non-receptor type 2 (PTPN2), specifically the 48 kDa isoform, which leads to the stimulation of the PI3K/Akt pathway, further promoting the IL-6 gene [63] (Figure 4).

#### 3.2.2. Proposed Mechanism: The Thrombin–FgDP–IL-6–TPO Pathway

Additionally, we propose a second pathway: the thrombin–FgDP–IL-6–TPO pathway. Thrombin catalyses fibrin formation and this subsequently leads to the release of FgDP and FbDP via fibrin(ogen)olysis. Since FgDPs have been shown to stimulate IL-6 release from monocytes, it is plausible that thrombin indirectly regulates TPO production via FgDP-induced IL-6 synthesis.

To summarise this theory: following thrombin catalytic conversion of fibrinogen into fibrin, subsequent fibrin(ogen)olysis by plasmin leads to the generation of FgDPs, including fragments D, E, and D-dimer. Beyond their classical roles in coagulation, FgDPs have been increasingly recognized for their immunomodulatory functions. Notably, FgDPs have been shown to stimulate pro-inflammatory cytokine release from monocytes and hepatic cells. Mandl et al. and more recently others [15,64], demonstrated that fibrinogen fragment D/FgDP/D-dimers significantly induces IL-6 production in perfused mouse livers and human monocytes, establishing a direct link between fibrin(ogen)olysis and cytokine synthesis. IL-6, in turn, is a potent stimulator of TPO synthesis by hepatocytes, as shown first by Kaser et al. [25] in 2001, who demonstrated that IL-6 infusion leads to marked upregulation of hepatic TPO mRNA and circulating TPO levels in mice. Whilst Wang et al. [65] recently reported that elevated IL-6 levels in pancreatic cancer patients stimulate hepatocytes to synthesize TPO, contributing to a hypercoagulable state. Their study highlighted a feedback loop involving IL-6 and the Notch signalling pathway, which further amplifies IL-6 production and TPO synthesis.

Collectively, these findings support the hypothesis that thrombin may indirectly regulate TPO production via an FgDP-induced IL-6 signalling axis, linking coagulation, inflammation, and thrombopoiesis in a feedback loop with potential relevance to both physiological haemostasis and inflammatory thrombocytosis.

While we focus on PAF and thrombin, it is important to note that other platelet-secreted molecules capable of inducing IL-6 could also play a role in this feedback mechanism. Further studies are needed to validate these pathways and their implications for platelet homeostasis and coagulation regulation.

**Figure 5:**
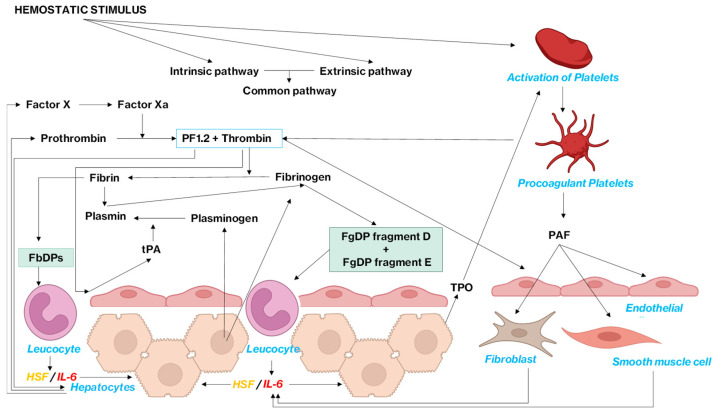
Complete overview. Both the intrinsic and extrinsic pathways contribute to the activation of coagulation factor X, which is converted into its active form, coagulation factor Xa. Factor Xa plays a crucial role by converting prothrombin into thrombin, and more significantly, generating PF1.2. PF1.2 acts directly on hepatocytes, stimulating the synthesis of both coagulation factor X and prothrombin. Thrombin then catalyses the conversion of fibrinogen into fibrin, leading to clot formation. Once the clot forms, it stimulates plasminogen activation into plasmin, which degrades fibrinogen, resulting in the formation of FgDPs. These degradation products interact with leukocytes, triggering the release of IL-6, which in turn induces fibrinogen, plasminogen and TPO synthesis in hepatocytes. This process is further enhanced by procoagulant platelet function. Following an endothelial lesion, collagen exposure will cause activation of procoagulant platelets. This will stimulate the release of two compounds: one being PAF and the second one being thrombin. The latter utilises prothrombinase complexes. FgDP and PAF will then stimulate the secretion of IL6; which in turn stimulates the release of TPO, fibrinogen and plasminogen. Created with BioRender. Legend: coagulation factor X (Factor X), coagulation factor X activated (Factor Xa), fibrin degradation products (FbDPs), fibrinogen degradation products (FgDPs), hepatocyte-stimulating factor (HSF), interleukin-6 (IL-6), platelet activating factor (PAF), prothrombin fragment 1.2 (PF1.2), tissue plasminogen activator (tPA), thrombopoietin (TPO).

All the above-described pathways, excluding the coagulopoietin and AMR pathway, can be visualised in Figure 5.

Experimental evidence suggests that the proposed feedback loops operate on biologically plausible timescales. Thrombin-induced tPA release and subsequent FgDP formation occur within hours [40,43,44], and FgDPs such as fragment D and D-dimer rapidly stimulate IL-6 secretion from monocytes and liver tissue in vitro and ex vivo [14,15,26]. Similarly, platelet-derived PAF induces IL-6 transcription in endothelial and stromal cells within 4–24 h [56,57,58,59,60,61,62,63]. In hepatocytes, IL-6 upregulates plasminogen and fibrinogen mRNA, with isoform-selective increases of ~3.6-fold for γA and ~8.3-fold for γ′ reported after 24 h [23,36,38], while also stimulating TPO mRNA and protein within 6–24 h [54]. In vivo, systemic IL-6 elevates hepatic TPO and circulating levels within a day, with platelet count changes following thereafter [25]. Physiological acute responses thus produce transient IL-6 rises and modest increases in fibrinogen, plasminogen, and TPO that restore haemostatic balance [20,23,24,36,38,54], whereas pathological conditions such as chronic inflammation or sepsis sustain higher IL-6 amplitudes, driving prolonged fibrinogen elevation (particularly γ′ isoform), reactive thrombocytosis, and feed-forward amplification via continued FgDP generation [7,22,25].

## 4. Biological and Medicinal Impact-Novel Anticoagulant and Anti-Thrombotic Drugs

Knowing and understanding the regulatory mechanisms of coagulation factors/thrombin generation is of major importance in the development of new therapeutic targets and drug-development in the case of older patients with comorbidities and polypragmasy where the ischemic/bleeding risk balance is extremely sensitive, but also in the case of young patients e.g., post-traumatic, post-COVID, etc. Therapeutic targets can include the ex vivo stabilization of platelets and the increase in their haemostatic function by blocking VWF binding with the ARC 1779/BAX 930 aptamer, or by counteracting the release of VWF from Weibel-Palade bodies through syntaxin-3 [66].

The use of direct thrombin inhibitors or PAR-1 and PAR-4 receptor blockers (protease activated receptor 1 and 4), especially the PAR-4-Thr120 variant responsible for approximately 50% of platelet hyperactivity, may represent promising translational candidates to replace treatment with unfractionated heparin [67]. Platelets can be stabilized by antagonizing collagen-induced platelet aggregation on GPVI (glycoprotein VI) receptors by ACT017 antibodies or fibrin fragment d dimer [66]. PAI-1 inhibitors favour fibrinolysis by removing the inhibitory effect of PAI-1 on the conversion of plasminogen to plasmin, a serine protease that degrades fibrin and several other proteins in plasma [68].

Anticoagulants that target factor XIa (IONIS-FXIRx, fesomersen, osocimab, abelacimab, milvexian, asundexian or xisomab 3G3) are currently under study as they inhibit thrombosis without impairing haemostasis [69]. Regarding therapy with oral anticoagulants, research is currently directed towards finding a universal anticoagulant reversal agent that would constitute an antidote for both heparins and oral anticoagulants; in this sense, andexanet alpha or ciraparantag, an anti-factor IIa and/or factor Xa compound, are already developed [70].

Classic antiplatelet drugs, acetylsalicylic acid, an irreversibly cyclooxygenase-1 (COX-1) inhibitor and suppressor of thromboxane A_2_-mediated platelet aggregation and clopidogrel, an ADP-mediated platelet aggregation inhibitor via P2Y_12_ receptor blockade have low or no efficacy in the novel thromboinflammatory mechanisms discussed in this work [71,72]. Procoagulant platelets, activated independently of the COX-1/TXA_2_ pathway are largely resistant to acetylsalicylic acid [49]. Furthermore, acetylsalicylic acid or clopidogrel do not modulate the PAF–IL-6–TPO axis implicated in reactive thrombocytosis does and does not affect the IL-6 induction by FgDPs [63,73]. Unlike the selective PAR antagonists, GPVI inhibitors, or IL-6 blockers, acetylsalicylic acid lacks the ability to discriminate between platelet subpopulations and may contribute to increased bleeding risk by broadly suppressing all platelet functions [72,74,75]. Reported high on-acetylsalicylic acid “resistance” varies by cohort and assay, typically ~7–20% in ischemic stroke or ~4.1–50% in peripheral arterial disease. Clopidogrel nonresponse is likewise frequent, e.g., ~30% in primary stroke and ~38% in recurrent stroke cohorts [76].

Newer P2Y_12_ inhibitors such as ticagrelor and cangrelor offer improved antiplatelet efficacy over clopidogrel but limited utility in thromboinflammatory conditions. Although ticagrelor may exhibit modest anti-inflammatory effects through adenosine reuptake inhibition, these are insufficient to modulate the cytokine-driven prothrombotic effects [77].

In this respect, newly proposed feedback pathways—particularly those involving FgDPs, IL-6, PAF, and TPO—reveal a dynamic interplay between coagulation, inflammation, and hepatic synthesis, which can be pharmacologically exploited. In patients with thromboinflammatory disorders, such as sepsis, cancer-associated thrombosis, or autoimmune vasculopathies, the dysregulated production of IL-6 and FgDPs may simultaneously promote a hypercoagulable state and maladaptive thrombopoiesis. Therapeutically, targeting cytokine-induced TPO synthesis or PAF-mediated IL-6 release could refine existing strategies by adding precision control over platelet homeostasis, in contrast to the broad anticoagulation strategies currently in use.

### 4.1. Targeting the IL-6-TPO Axis

Clinically, PAF–IL-6–TPO pathway is particularly relevant in conditions of reactive thrombocytosis, such as sepsis, autoimmune vasculopathies, and cancer-associated thrombosis, where excess platelet production exacerbates thrombotic risk [2,78]. Current antiplatelet drugs, including aspirin and clopidogrel, fail to specifically target procoagulant platelet subpopulations or cytokine-driven TPO upregulation. By contrast, modulation of the PAF–IL-6–TPO axis offers a potential means of selectively controlling maladaptive thrombopoiesis while preserving physiological haemostasis.

Inhibitors of IL-6 signaling like tocilizumab are already in clinical use for cytokine release syndromes, rheumatoid arthritis and COVID-19-associated cytokine storm [27,28]. Given the data linking IL-6 to TPO upregulation, these agents may have off-label potential in controlling reactive thrombocytosis, especially in malignancies or inflammatory vascular syndromes [65]. Although IL-6 blockade by tocilizumab or sarilumab is a rational way to decrease cytokine-driven TPO induction, it carries class-specific risks such as increased incidence of bacterial infections, blunts fever and CRP, potentially masking infection and elevated liver enzymes, neutropenia, thrombocytopenia, and lipid increases are also described [79]. The use of IL-6R inhibitors in rheumatoid arthritis shows increased risk of gastrointestinal perforation, particularly in patients with diverticular disease and/or on concomitant NSAIDs or corticosteroid treatment [80].

Beyond their anti-inflammatory effects, IL-6 blockers reduce downstream acute-phase signalling like STAT3 activation, providing a mechanistic basis for attenuating TPO-driven platelet overproduction [81]. Recent clinical trials and living meta-analyses in hyperinflammatory conditions confirm that IL-6 blockade is safe and modulates key inflammatory readouts, supporting translational exploration in cytokine-driven thrombocytosis [24,82].

Furthermore, evidence shows that hepatocytes are highly sensitive to low IL-6 concentrations, suggesting a threshold-dependent transcriptional activation of TPO synthesis [24]. Mechanistically, IL-6 engages classic or trans-signalling (IL-6Rα/sIL-6R with gp130) to activate JAK-STAT3 in hepatocytes. At sub-threshold IL-6 levels, STAT3 phosphorylation and promoter occupancy remain insufficient for meaningful transcription; above a functional threshold, cooperative STAT3 recruitment and co-activator engagement produce a non-linear rise in acute-phase gene expression, followed by SOCS3-driven negative feedback that limits signal duration. This threshold-then-plateau behaviour aligns with hepatocyte biology, where even relatively low IL-6 exposures can trigger acute-phase programs, and provides a rationale for IL-6 blockade to blunt cytokine-driven thrombopoietin induction in reactive thrombocytosis [83,84,85].

In thromboinflammatory disease, where IL-6 often co-rises with fibrin(ogen) degradation products and endothelial activation, targeting IL-6 may simultaneously mitigate coagulopathy, vascular inflammation, and secondary thrombocytosis [7].

### 4.2. Targeting the Thrombin–FgDP–IL-6–TPO and FgDP–IL-6–Plasminogen Pathways

The fibrinolytic degradation products, particularly those derived from fibrinogen, have emerged as key regulators not only of coagulation turnover, but also of immune signaling and hepatic biosynthetic pathways [2,7]. These mechanistic insights open new therapeutic possibilities for conditions marked by thromboinflammatory dysregulation, including sepsis, malignancy-associated coagulopathies, and autoimmune vasculitis [65].

One promising strategy involves the development of FgDP-mimicking compounds designed to selectively stimulate hepatocyte-driven regeneration following coagulopathic injury, while minimizing the risk of systemic inflammation or prothrombotic complications. To minimize off-target monocyte/macrophage activation, ‘FgDP-mimicking’ agents should be designed as hepatocyte-restricted endosomal/pH or enzyme-activated prodrugs, with liver-targeted delivery, such as GalNAc–ASGPR conjugates or liver-tropic nanoparticles and epitope editing to remove β2-integrin–binding motifs implicated in macrophage IL-6 induction by fibrin(ogen) fragments [86,87]. Recombinant analogues of fragment D or fragment E could be engineered with liver-targeting moieties to optimize tissue specificity and therapeutic efficacy [24].

On the other hand, the excessive release of IL-6 in response to FgDP-monocyte interactions may exacerbate hypercoagulability, indicating a role for targeted inhibition of the FgDP–IL-6 axis. Therapeutic candidates include IL-6 receptor antagonists (such as tocilizumab), NF-κB inhibitors, and integrin p150,95 (CD11c/CD18) blockers, all of which may disrupt pro-inflammatory signaling cascades initiated by fibrinolytic by-products [63].

In parallel, dual-pathway modulators that exert both anticoagulant and anti-inflammatory effects are gaining interest. For example, statins and fibrates, beyond their lipid-lowering properties, have demonstrated the ability to attenuate IL-6 and CRP expression, reduce platelet activation, and modulate vascular inflammatory tone [64,88,89]. Statins and fibrates have direct lipid-modifying actions (statins: ↓LDL/ApoB; fibrates: ↓TG/VLDL, ↑HDL) and pleiotropic effects on inflammation, endothelium, and haemostasis. Through reduced IL-6/CRP, PAI-1/TF down-modulation, improved NO bioavailability, and lower platelet reactivity, these agents can blunt IL-6–dependent hepatic outputs and favour fibrinolysis. Thus, beyond lipid control, their pleiotropy provides a mechanistic rationale for modulating the FgDP–IL-6 axis in thromboinflammatory states [90,91].

Additionally, therapeutic combinations such as rivaroxaban with colchicine, or emerging IL-6-targeting peptides, are under investigation in the context of thromboinflammatory disorders [92]. Hence, current understanding of the FgDP–IL-6–TPO and PAF–IL-6–TPO pathways raises the possibility of selectively modulating thrombopoiesis at the level of inflammatory signalling rather than interfering with core haemostasis. Procoagulant platelets release platelet-activating factor (PAF) and microparticles, which potently induce IL-6 production in endothelial cells, fibroblasts, and vascular smooth muscle cells [56,57,58,59,60,61,62]; thus, PAF receptor (PAFR) antagonism could attenuate IL-6 induction without impairing aggregation via COX-1 or P2Y12. Similarly, fibrinogen degradation products (FgDPs), particularly fragment D and D-dimer, stimulate IL-6 release from monocytes and liver tissue [15,26], and targeting the integrin p150,95 (CD11c/CD18) or downstream NF-κB pathways may reduce this signal without disrupting thrombin generation or primary clot formation [14,17,64]. Notably, statins and fibrates have been shown to blunt FgDP-induced IL-6 in vascular cells, suggesting repurposable anti-inflammatory strategies that spare coagulation [64,88,89]. At the cytokine level, IL-6 receptor (IL-6R) blockade has been shown to decrease TPO upregulation and reactive thrombocytosis [25,27,28]. Because basal TPO production is also maintained through Ashwell–Morell receptor (AMR)–JAK2–STAT3 signalling from desialylated platelets [52,55], IL-6R inhibition would be expected to preferentially blunt the reactive, inflammation-driven component of thrombopoiesis while preserving the physiological platelet turnover mechanism. In contrast, broad antiplatelet agents such as aspirin or P2Y12 inhibitors are ill-suited to this purpose, as they do not target the IL-6 axis and carry a risk of bleeding complications [71,72,73,77]. Together, these considerations support the feasibility of dampening cytokine-driven platelet production in inflammatory and thromboinflammatory states through selective targeting of the FgDP–IL-6–TPO and PAF–IL-6–TPO pathways, while maintaining the integrity of haemostatic and immune functions. Collectively, these strategies signify a paradigm shift toward integrated modulation of the coagulation-inflammation interface, offering the potential for more nuanced and disease-specific interventions in patients with disrupted hemostatic and immune balance.

### 4.3. Platelet Subpopulation-Specific Therapies

While aggregatory platelets mediate physiologic plug formation via αIIbβ3-dependent aggregation and are the principal targets of aspirin and P2Y_12_ inhibitors., procoagulant platelets, a necrotic-like subset generated under strong GPVI/PAR co-stimulation, exhibit sustained Ca^2+^, mitochondrial depolarization, phosphatidylserine exposure, robust thrombin generation, and PAF release; they are relatively resistant to aspirin and can amplify IL-6–linked feedback to TPO, thereby propagating thrombo-inflammation [93]. Accordingly, therapies that act upstream on the procoagulant program, such as PAR-4 (±PAR-1) antagonists, GPVI inhibitors, PAF-receptor antagonists, and downstream IL-6 pathway inhibitors, are mechanistically suited to dampen the procoagulant phenotype while preserving baseline aggregation and hemostasis [94].

Procoagulant platelets, characterized by phosphatidylserine exposure and potent thrombin generation, contribute to thrombotic pathologies, whereas aggregatory platelets are primarily involved in physiological clot formation and retraction [49]. Therapeutic strategies aim at limiting thrombosis while preserving haemostasis. In this context, agents that inhibit PAF synthesis or block PAF receptors (PAFR) may reduce IL-6-driven TPO upregulation, thereby controlling reactive thrombocytosis without impairing platelet aggregation [63]. Beyond PAF modulation, several receptor-specific therapies show promise in targeting pathological platelet activation. These include PAR-1 and PAR-4 antagonists such as vorapaxar or BMS-986120 [95], which inhibit thrombin-induced platelet activation; GPVI inhibitors (e.g., ACT017) [96], which block collagen-mediated platelet responses; and PAI-1 inhibitors, which enhance endogenous fibrinolysis [97].

The mechanisms underlying conventional and novel (targeted) therapeutic approaches are compared in Table 1.

## 5. Conclusions

Recent advances in platelet biology, coagulation dynamics, and TPO regulation have significantly expanded our understanding of the complex feedback mechanisms governing haemostasis. Initially, TPO homeostasis was thought to be solely dependent on platelet-mediated uptake and clearance, where excess platelets reduce circulating TPO levels and platelet deficiency increases its availability [51]. However, emerging research has challenged this classical view, demonstrating that aged platelets actively stimulate TPO synthesis via the AMR on hepatocytes, linking platelet turnover to hepatic thrombopoiesis through a receptor-mediated mechanism [52].

Novel findings have highlighted the critical role of platelet subpopulations in coagulation and inflammatory signalling. The distinction between procoagulant platelets and aggregatory platelets has shed light on differential platelet functions beyond clot formation, including their role in thrombin generation, necrosis, and cytokine signalling [49,98]. The ability of procoagulant platelets to secrete PAF, subsequently inducing IL-6 synthesis across multiple cell types, forms the foundation of our proposed PAF–IL-6–TPO pathway, which hypothesizes that this signalling ultimately enhances hepatic TPO synthesis as a means of compensating for platelet consumption following coagulation [56,59,60].

Mechanistic evidence already supports key elements of the proposed FgDP–IL-6–TPO axis. Several studies have demonstrated that FgDPs, particularly fragment D and D-dimer, induce IL-6 secretion in human monocytes and in perfused murine livers [15,26], while fragment E has also been shown to stimulate IL-6 production in rat macrophages [14]. In parallel, the downstream effect of IL-6 on hepatic TPO production is well established: IL-6 directly increases TPO mRNA and protein expression in hepatocyte-derived cell lines [54] and elevates circulating TPO levels in both murine models and human subjects [25]. Although no single experimental system has yet demonstrated the entire cascade from FgDPs through IL-6 to TPO synthesis, the two critical mechanistic links are each experimentally validated. This strongly supports the biological plausibility of our hypothesis that fibrinolytic by-products can regulate thrombopoiesis via an IL-6–dependent hepatic pathway.

Hence, furthermore, we propose the thrombin–FgDP–IL-6–TPO pathway, in which FgDP-induced IL-6 secretion may serve as a feedback mechanism linking fibrinolysis to thrombopoiesis and regulating TPO levels in response to platelet consumption [51,52]. In addition, it is crucial to elucidate the role of APC in the release of tPA and to further investigate whether coagulopoietins physiologically compensate for vitamin K deficiency.

Collectively, these insights introduce a paradigm shift in understanding platelet regulation, coagulation, and thrombopoiesis. Instead of being passive elements in haemostasis, platelets actively participate in feedback loops that regulate their own production, involving a complex interplay of coagulation-derived messengers, cytokines, and hepatic response mechanisms. These findings provide initial guidelines for potential future precision-guided therapies that modulate platelet–cytokine feedback loops in thromboinflammatory disorders.

## Figures and Tables

**Table 1:** Conventional vs. novel interventions across IL-6–TPO, thrombin–FgDP–IL-6–TPO, FgDP–IL-6–plasminogen and platelet subpopulations.

**Target**	**Goal**	**Conventional Therapies (Mechanism)**	**Novel/Targeted Therapies (Mechanism)**	**Potential Risks**
IL-6–TPO axis	Blunt cytokine-driven thrombopoiesis (reactive thrombocytosis). ↓IL -6 signaling → ↓TPO output; potential ↓fibrinogen; platelets trend toward normalization	-Corticosteroids (broad ↓cytokines incl. IL-6); -NSAIDs (minimal on IL-6/TPO); -general anticoagulants (heparin/DOACs/VKA) have no direct effect on IL-6/TPO	-IL-6R mAbs (tocilizumab, sarilumab → block classic/trans-signaling); -JAK inhibitors (e.g., baricitinib → dampen STAT signaling); -PAF-R antagonists (upstream ↓IL-6); statins/fibrates (pleiotropy: ↓IL-6/CRP, ↓PAI-1)	-Bacterial infection by IL-6/JAK blockade; -IL-6 blockers may mask fever/CRP; lipid changes;
Thrombin–FgDP–IL-6–TPO axis	Reduce thrombin generation and/or IL-6 amplification from FgDP ↓FgDP formation and/or ↓IL-6 response → ↓TPO; improved inflammatory tone	-Heparins/DOACs/VKA (↓thrombin → ↓fibrin → ↓FgDP); -antifibrinolytics (TXA) ↓FgDP but may stabilize clots.	-PAR-1/4 antagonists (↓thrombin-platelet signaling); GPVI inhibitors (↓collagen-driven procoagulant platelets → less thrombin/PAF); -IL-6 pathway inhibitors (block downstream amplification)	-Anticoagulant/antiplatelet bleeding risk; -TXA may worsen thrombosis.
FgDP–IL-6–plasminogen axis	Tune fibrinolysis recovery without excessive IL-6 surge. Balanced: maintain fibrinolysis while preventing IL-6 overshoot; stable plasminogen pool	-tPA/tenecteplase (↑plasmin, ↑FgDP use for lysis, but may ↑IL-6); -PAI-1↑ (physiologic/inflammatory) dampens fibrinolysis; -fibrates (↓PAI-1).	-PAI-1 inhibitors (pro-fibrinolytic; may ↑FgDP → watch IL-6); -IL-6 blockade to cap cytokine surge during high FgDP states; -hepatocyte-targeted FgDP-mimic without leukocyte activation	-β2-integrin/TLR activation on monocytes must be avoided
Platelet subpopulation–specific (procoagulant vs. aggregatory)	Selectively dampen procoagulant platelets while preserving physiologic aggregation. ↓PAF and thrombin-driven IL-6 triggers → indirect ↓TPO; less propagation of thrombo-inflammation.	-Aspirin/P2Y12 inhibitors reduce aggregation but do not specifically target procoagulant phenotype	-PAR-4 (±PAR-1) antagonists (thrombin-platelet axis); -GPVI inhibitors (collagen axis); -PAF-R antagonists (↓PAF-driven IL-6).	-Bleeding risk

Abbreviations: FgDP—fibrinogen degradation products; PAF—platelet activating factor; PAF-R—PAF receptor; DOAC—direct oral anticoagulant; VKA—vitamin K antagonist; TXA—tranexamic acid; IL-6R mAb—interleukin-6 receptor monoclonal antibody; GPVI—glycoprotein VI. ↓ down arrows mean decrease and ↑ up arrow mean increase.

## Data Availability

Data supporting the findings of this study can be requested from the corresponding author (M.S.) upon reasonable justification.
